# Clinton Co. Medical Society, Iowa

**Published:** 1872-04-15

**Authors:** 


### Society Reports.

### CLINTON CO. MEDICAL SOCIETY, IOWA.

The Clinton County Medical Society held their regular quarterly meeting at Dr. Morgan's office in DeWitt, April 16, 1872. The meeting was called to order by the President, Dr. D. Langan, at 5 o'clock p. m. Charles H. Lothrop was appointed Secretary *pro tem.* The roll was called, and showed the following members present: Drs. Langan, Lothrop, Frost, Morgan, Dennison, and Prof. Farnesworth. The minutes of the previous meeting were read and adopted. The application of Dr. Willis Butterfield for membership was received and accepted, and on motion of Dr. Farnesworth he was requested to take part in the proceedings of the present meeting. The Society then adjourned for supper, accepting the invitation of Dr. Langan, and a couple of extremely pleasant hours were passed in the enjoyment of his hospitality.

Business was again resumed at 8 o'clock; and a very able paper was read by Dr. Morgan on "Fractures of the Femur, and treatment," which was fully discussed by Drs. Farnesworth, Frost, Butterfield and Lothrop. Next Prof. Farnesworth read a scholarly paper on "Medical Education," which received the approbation of the Society. On motion, a committee was appointed to report to the State Society on the New York law on malpractice, which requires the plaintiff to give bonds in twice the amount of the damages claimed. Dr. Langan appointed Prof. Farnesworth, Dr. Lothrop, and Dr. Morgan.

The following members were appointed to read essays at the next meeting: Dr. Charles C. Lothrop, *Cerebro-Spinal Meningitis;* Dr. J. Dennison, *Puerperal Fever;* Dr. Frost, *Scarlet Fever;* Dr. Reynolds, *Typhoid Fever.*

On motion of Prof. Farnesworth, Dr. Langan was appointed the delegate of the

Clinton County Medical Society, to the American Medical Society, which is to meet at Philadelphia in May, 1872. Quite a lively discussion upon cerebro-spinal meningitis, which was participated in by Drs. Dennison, Morgan, Lothrop, and Farnesworth; and the symptoms, pathology, and treatment were fully discussed, inasmuch as the disease has lately attracted much attention in this locality, as well as in other parts of the United States.

The meeting was one of great interest, and the Society remained in session until a late hour at night, when they adjourned, to meet in Camanche on the third Tuesday in July.

